# Supplementary material for: Identification of a Novel Protein-Based Prognostic Model in Gastric Cancers
Source: Biomedicines. 2023 Mar 22;11(3):983. doi: 10.3390/biomedicines11030983 (PMC10046574; doi:10.3390/biomedicines11030983)
Supplement: Supplementary file 1 [file biomedicines-11-00983-s001.zip › biomedicines-2137356-supplementary.pdf]

Supplementary Table S1. The clinical information of the GC cohort

Clinicopathological features	Levels	Number (%)
Age (years)		65.25 ±10.89
Gender, *n* (%)	Female	130 (36.9%)
	Male	222 (63.1%)
Pathological diagnosis	Adenocarcinoma	352 (100%)
T stage, *n* (%)	T1	12 (3.4%)
	T2	69 (19.6%)
	T3	169 (48%)
	T4	102 (29%)
N stage, *n* (%)	N0	104 (29.6%)
	N1	94 (26.7%)
	N2	74 (21%)
	N3	80 (22.7%)
M stage, *n* (%)	M0	325 (92.3%)
	M1	27 (7.7%)
Pathologic stage, *n* (%)	Stage I	39 (11.1%)
	Stage II	106 (30.1%)
	Stage III	167 (47.4%)
	Stage IV	40 (11.4%)
Tumor grade, *n* (%)	G1	7 (2%)
	G2	119 (33.8%)
	G3	226 (64.2%)
